# The State Board of Medical Examiners

**Published:** 1883-03

**Authors:** 


### Editorial.

### THE STATE BOARD OF MEDICAL EXAMINERS.

The meeting of the New York State Medical Society, which held its annual session at Albany, last month, was for many reasons the most important, as it was the most interesting of any in the history of that society. We affirm this, not having in view the thoroughly scientific and scholarly papers which were presented, and the clear and able discussion which they called forth, as much as the action of the society, after an animated debate, on the ethical and educational questions which occupied so much of the time and attention of the delegates.

There was an unusual interest in the general business of the session as was apparent in the large attendance of delegates and members, while the unlooked for result of the discussion upon the question of the New Code, which was adopted a year ago and reaffirmed at this session, after a very exciting debate, marks an epoch in the history of the medical profession in this State, which claims our serious consideration.

As widespread as has been the interest excited by the State Society on the position it assumed a year ago on the subject of Medical Ethics, in our opinion, the most vital question presented for the consideration of the society, was the recommendation of a bill for the creation of a State Board of Examiners. The proposed bill came before the society in the report of the committee on legislation, and was evidently prepared with great care. Its sets forth:

1st. What it means to be a practicing physician in this State.

2d. That after some future time (Dec. 1883) a person shall not have the right to practice medicine in this State unless licensed by the State Board of Examiners.

3d. That candidates for license to practice medicine may select to be examined in any of the schools of practice represented by the incorporated State medical societies.

4th. That the records of the examinations, upon which licenses are granted, shall remain a part of the archives of the University of the State.

5th. It repeals all acts or parts of acts authorizing medical colleges or other corporations to grant degrees which shall be licenses to practice medicine.

There can be no question in the minds of disinterested persons as to the absolute necessity of the enactment of a law which embodies such important principles as is set forth in this bill. We are happy to give the matter our most unqualified approval and support. Our readers will recall the position we have heretofore taken upon the subject of medical education, and the JOURNAL is more emphatic than ever in its advocacy of measures which will repair the lamentable weakness of our present system by placing the authority to grant diplomas and licenses in the hands of a Board, composed of the ablest representatives of the profession, who have no interest, direct or indirect, in bestowing what should be a coveted honor, except the the honorable ambition to elevate and improve the science of medicine through a wise choice of its votaries.

The fatal weakness of the present system of granting diplomas, which are really licenses to practice medicine, is its relation with the individual and pecuniary interests of those upon whom this authority now rests. Private corporations have no business with such responsibilities. It should be the prerogative of medical schools to teach and instruct only, and their chief ambition and aim should be to present thoroughly qualified candidates for the degree of Doctor of Medicine, to such an independent Board as the bill contemplates, which shall sit in judgment upon their work.

It would be idle to deny that from the day of the establishment of the first medical college until the present time there has been a widespread distrust among the profession of the methods and principles upon which they have been conducted. It is impossible to separate the individual, however high and noble may be the aims of such an one, from the work in which he may be engaged, especially if such a work adds to his private gains or to his professional reputation. As long as the educating power and the authority to license are vested in the same body this distrust will prevail and abuses will creep in. This bill proposes to separate them, and to endow a body entirely disconnected with the schools, with the power to sit in judgment on their labors. Can any fail to foresee the outcome of this important movement, that the law of natural selection will soon determine, not only the material for the future of the profession, but those also whose lives will be devoted to the noble work of medical educators.

What will the medical schools do in regard to this bill? If even in a quiet way they try to defeat the enactment of so wise a law as is here contemplated, such a course would be a lamentable confession of their fear that at least a portion of their work will not bear the closest scrutiny. Rather do we hope that wiser counsels will prevail, and that the inevitable will be accepted by them in their acquiescence in measures which will elevate the standard of medical education, and endow their medical degrees with positive value.

